# Validation of an HPLC Method for Determination of Bisphenol-A Migration from Baby Feeding Bottles

**DOI:** 10.1155/2019/1989042

**Published:** 2019-03-28

**Authors:** Ruth Rodriguez, Elianna Castillo, Diana Sinuco

**Affiliations:** ^1^Laboratorio de Extensión y Asesorías, Departamento de Química, Universidad Nacional de Colombia, Bogotá, Colombia; ^2^Grupo de Estudios para la Remediación y Mitigación de Impactos Negativos al Ambiente (GERMINA), Departamento de Química, Universidad Nacional de Colombia, Bogotá, Colombia; ^3^Grupo Bioprospección de Compuestos Volátiles, Departamento de Química, Universidad Nacional de Colombia, Bogotá, Colombia

## Abstract

A simple and economic high-performance liquid chromatography (HPLC-UV-Vis) analytical method was validated for the quantitation of specific Bisphenol-A migration from baby feeding bottles. Overall and specific migration assays were done with different food simulating matrices using the filling method. Good linearity was obtained over the concentration range of 0.01–0.6 mg/kg. The limit of detection (LOD) and limit of quantification (LOQ) were 0.004 and 0.010 mg/kg, respectively. The repeatability of the method (%RSD, *n*=10) was between 89.5 and 99.0%, while recovery ranged from 83.2 to 98.4%. The method was applied to specific migration assays from baby feeding bottles purchased from different plastic producers in Colombia. The results show that, in a first migration assay, Bisphenol-A was not detectable in all samples. In a second migration test, Bisphenol-A concentrations were higher than the most restricted limit (0.05 mg/kg) with ethanol 95% and isooctane as food simulants.

## 1. Introduction

Plastic food packaging or containers are produced with the help of plasticizers or additives used in plastic manufacture to improve its flexibility, stability, or resistance properties. Among these additives, 2,2-bis(4-hydroxyphenyl)-propane (Bisphenol-A) is a monomer used for the synthesis of polycarbonate (PC) and as a reaction intermediate in the manufacture of epoxy resins, phenoxy resins, polysulfone resins, and certain polyester resins, as well as flame retardants and in the manufacture of rubber [1]. Although its use is authorized by Commission Regulation [2], a prohibition is in place on its use in the manufacture of polycarbonate infant feeding bottles on the basis of the precautionary principle [3]. Bisphenol-A is part of the group of endocrine-disrupting compounds that interfere with the biosynthesis of hormones, metabolism, and the actions resulting from these, causing an alteration in the normal homeostasis of the exposed individual or his descendants. It has been shown that Bisphenol-A has an affinity for estrogen receptors and, therefore, possesses the ability to produce estrogenic effects [4, 5]. These additives could migrate into the food presenting a risk for human health. In this order, some regulations have been developed to establish the maximum overall migration limit (OML) and specific migration limit (SML).

The overall migration assays are based on gravimetric determinations, using different food simulants, which are in contact with the packaging material, under certain conditions of temperature, time, and exposition [6, 7]. The OML for plastic in contact with food is 50 mg/kg [8].

Specific migration assays are done by direct analysis of food simulants after finishing the overall migration conditions. For Bisphenol-A, a prevalidated HPLC-UV-Vis method is capable of quantitative determination as a minimum level of 0.2 mg/kg of food simulants [9]. Recently, a hardener restriction on the migration into or onto food of 2,2-bis(4-hydroxyphenyl)propane from varnishes or coatings applied to materials and articles has not exceeded a SML of 0.05 mg of Bisphenol-A per kg of food [3].

Bisphenol-A migration studies on baby feeding bottles has been conducted using derivatization and analysis by GC-EI/MS/MS [10], by GC-MS operated in the selected ion monitoring mode [11], by ultra-high-performance liquid chromatography (UPLC) with a fluorescence detector [12], and by stable-isotope dilution liquid chromatography-tandem mass spectrometry [13] among others. Although these studies offer very low detection limits (*µ*g/kg), for routine analysis and in accordance with regulation on SML, a simple, fast, economic, and reliable chromatographic method must be validated.

In Colombia, several plastic industries require the analysis of specific migration of Bisphenol-A. The “Extension and Counseling Laboratory” of Universidad Nacional de Colombia is an accredited lab which offers overall and specific migration assays, using an economic and reliable methodology. In this work, we validate the method by HPLC-UV-Vis to quantify specific migration of Bisphenol-A. Validation of the proposed method was performed in terms of selectivity, linearity, LOD and LOQ, precision, and trueness. Several baby feeding bottles purchased from different local producers were analysed in order to demonstrate the applicability of the validated method.

## 2. Materials and Methods

### 2.1. Samples

A total of 30 baby bottle samples were used for the determination of Bisphenol-A. The samples came from different companies within Colombia. One company supplied PC colour baby feeding bottles, one company supplied colourless PC baby feeding bottles, and another company supplied Bisphenol-A-free baby feeding bottles. Respecting a confidentiality agreement, the manufacturers are not mentioned.

### 2.2. Reagents and Materials

Standards of Bisphenol-A (CAS 80-05-7, purity ≥99%) were purchased from Sigma-Aldrich (St. Louis, Missouri, USA). Purified water was obtained with a Milli-Q Plus system (Milli-Q®, Millipore, Billerica, MA, USA). HPLC grade methanol, ethanol 95%, acetic acid, and isooctane were purchased from Merck and Panreac (Germany). Filters were PTFE Acrodisc® Syringe 13 mm, 0.2 *μ*m pore size GHP from Waters (USA). Stock solution of Bisphenol-A was prepared in methanol and stored at 4°C in the dark, with an expiry date of one week. For the cleaning of alternative fatty food simulants, a mixture of water : methanol 1 : 1 (Merck-Germany) and hexane (Panreac-Germany) HPLC analytical grade was used.

### 2.3. Instrumentation

Liquid chromatographic analyses of Bisphenol-A were performed using a Hitachi Primaide 1120-system liquid chromatograph (Hitachi®, Palo Alto, CA) equipped with a quaternary high-pressure pump (1110 series), a single wavelength UV detector (1410 series), and an autosampler (1210 series). Data analysis was carried out using Hitachi ChemStation software. Chromatographic separations were developed on a Kinetex® C18 column (2.6 *μ*m, 4.6 × 100 mm) under an isocratic program using 50% of methanol (solvent B) and 50% of water (solvent A) as mobile phase components. The flow rate was set at 0.5 mL/min, the injection volume was 10 *μ*L, the analyte was monitored at 224 nm, and total analysis was done in 13 minutes. This method is an adaptation of a prevalidated method in UNE-EN: 13130-13:2005 [9]. Because the prevalidated method use different conditions, it is necessary to validate the method.

### 2.4. Method Performance

#### 2.4.1. Selectivity

In order to evaluate selectivity of the method, the analysis of blank samples (free Bisphenol-A baby feeding bottles) was done. The analysis of spiked blanks allows to identify Bishphenol-A signal and retention time.

#### 2.4.2. Working Range, Linearity, Limit of Detection (LOD), and Limit of Quantitation (LOQ)

Taking into account a SML for Bisphenol-A as 0.05 mg/kg, the linearity of the method was tested using standard solutions at eleven concentration levels equally distributed between 0.01 and 0.6 mg/kg. Each concentration level was analysed in triplicate, and the calibration curve was plotted using the average of area versus known concentration. Linearity evaluation was done by the analysis of relative standard deviation of the slope (%Sb), *y*-residuals, correlation coefficient (*r*^2^), and ANOVA lack of fit model (LOF) at a confidence level of 95% [14, 15].

The LOD and LOQ were calculated from the signal-to-noise ratio of chromatograms for blank samples (S/N = 3 for LOD and S/N = 10 for LOQ) and then expressed in concentration through the relation with the signal-to-noise ratio of a 0.01 mg/kg spiked blank [16].

#### 2.4.3. Accuracy

The accuracy of the test was determined in terms of precision (repeatability) and trueness (recovery).

The method precision (repeatability) was evaluated by analysing ten independent replicates at three different concentration levels over the working range of the method in the same day (intraday). Repeatability was expressed as percent relative standard deviation (%RSD).

The method trueness was evaluated on recoveries, measured after spiking samples of food simulants obtained after migration process with Bisphenol-A standard at different three different levels: 0.05 mg/kg, 0.3 mg/kg, and 0.6 mg/kg in different days (interday). Recovery percentages (%*R*) were calculated according to the following equation:

(1) $$\begin{array}{c} \%R=\left[\frac{\left(C_{1}-C_{2}\right)}{C_{3}}\right]\;*\;100, \end{array}$$

where *C*_1_ (mg/kg) is the concentration of Bisphenol-A determined in the fortified samples, *C*_2_ (mg/kg) is the concentration of Bisphenol-A in samples before spiking, and *C*_3_ (mg/kg) is the concentration of Bisphenol-A added to the sample.

### 2.5. Migration Tests

Overall filling method and specific migration assays were performed on 30 baby bottle samples, according to regulation [7, 9]. Briefly, each bottle was filled with 200 ml of food simulants, respectively. Three feeding bottles were subjected to the migration test with each food simulant: distilled water, acetic acid solution 3% (p/v), ethanol 15% (v/v), isooctane, and ethanol 95% (v/v), also called alternative fatty food simulants. Two control (Bisphenol-A-free baby feeding bottles) were also submitted to migration test. For aqueous, acidic, alcoholic, and ethanol 95%, the migration tests were done at 40°C during 10 days, and for isooctane, the assay was done at 20°C for 2 days.

For analysis of Bisphenol-A specific migration, 10 *μ*l of each food simulant was injected directly into the HPLC system without any additional sample treatment. All migration tests were performed in triplicate, and the results of the measurement area were correlated with an external calibration curve from 0.01 to 0.6 mg/kg. In order to simulate the possible Bisphenol-A migration due to reuse of feeding baby bottles, a second migration assay was done under the above-described conditions.

## 3. Results and Discussion

### 3.1. Method Validation

#### 3.1.1. Selectivity

**Figure 1:**
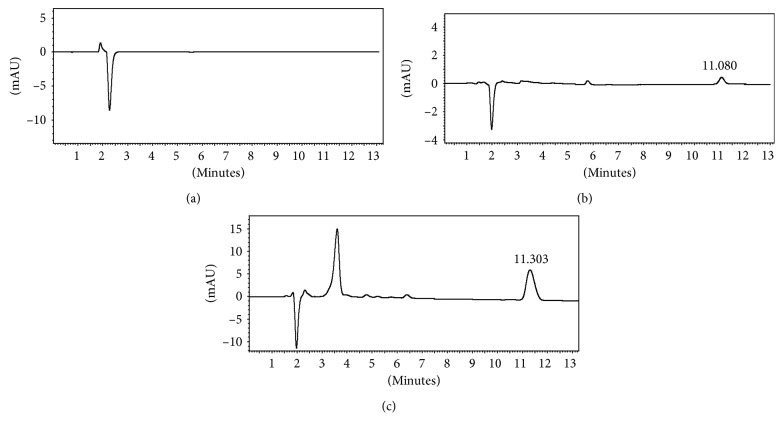
HPLC chromatograms of blank (a), Bisphenol-A standard solution (0.05 mg/kg) (b), and baby feeding bottle migration sample with ethanol 95% (c).

Selectivity was evaluated by comparison of chromatograms of blank (Bisphenol-A-free feeding bottles), Bisphenol-A standard solution, and the migration results from baby feeding bottle samples with all food simulants. In Figure 1, the analysis of overall migration assay with ethanol 95% as a food simulant is shown as an example.

Bisphenol-A signal can be observed at 11.080 min for the analysis of standard solution and at 11.303 min for migration sample. For specific migration analysis, the obtained Bisphenol-A peak is sharp, symmetrical, and completely separated from other analyte peaks. No interferents or coeluted peaks were observed for anyone of food simulants at the retention time for Bisphenol-A.

#### 3.1.2. Working Range, Linearity, Limit of Detection, and Limit of Quantification

**Figure 2:**
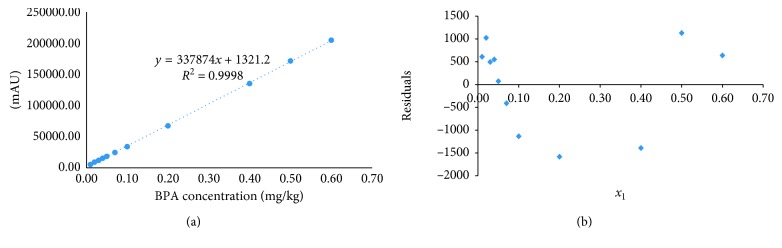
Standard calibration curve of Bisphenol-A by HPLC-UV-Vis and residual plot for 0.01 to 0.6 mg/kg interval.

Working range was selected according to the SML of 0.05 mg/kg. Linearity was demonstrated by analysing the Bisphenol-A solution in a concentration range of 0.01 to 0.6 mg/kg.  Figure 2 shows the results for the calibration curve and their residual plot.

From the regression analysis, a linear relationship over the concentration range of 0.01–0.6 mg/kg was obtained (*y* = 337874*x* + 1321) with a correlation coefficient of 0.9998. The residuals average was lower than 2%, and the relative standard deviation of the slope was 0.45%. These results are in agreement with JRC guidelines [15]. Additionally, in this work, ANOVA lack of fit model (LOF) was used. This model is based on the comparison of the tabulated *F* of Fisher values with the observed *F* of Fisher calculated on the basis on the experimental results and on the sums of squares. In this case, observed *F* calculated values were greater than tabulated *F* of Fisher values, and the linear regression model adequately fit the data (*F*_cal_ 6356 > *F*_tab_ 4.116). Therefore, the linearity was successfully verified over the working range employed.

The LOD (0.004 mg/kg) and LOQ (0.010 mg/kg) reached by this validated method are enough to ensure compliance even with hardeners international regulation. LOQ obtained in this study is comparable with that from a fluorescence detector (0.0098 mg/kg) [17], but it is higher than that obtained from a MS/MS detector (0.0033 mg/kg) [18]. However, not all labs can afford the cost of those specialized analysis. Also, the methodologies include clean-up steps, which involve longer time analysis. For those reasons, the validated method can be considered as suitable for routine Bisphenol-A migration analysis.

#### 3.1.3. Accuracy

Table 1 shows the results of the accuracy of the method in terms of precision (%RSD) and trueness (%recovery).

The repeatability was evaluated by analysing ten independent replicates of Bisphenol-A solutions at three concentration levels over the working range of the method. The relative standard deviation for lowest and intermediate concentration levels was higher than 95% and around 90% for highest concentration. A similar behaviour was observed in terms of recovery: the efficiency of the method varies according to the analyte concentration. For that reason, it is recommended to perform assays at three or two calibration levels [19]. In this work, three levels of concentration were evaluated, including the lowest specific migration limit of 0.05 mg/kg [3]. The results of accuracy showed percentage recovery at three levels in the range of 83.2–98.4%. These values are within the accepted limits, which indicate the applicability of the method for Bisphenol-A analysis.

#### 3.1.4. Migration Studies on Baby Feeding Bottles

The food simulants obtained after overall migration assays from baby feeding bottles were analysed by the validated method.  Table 2 shows the results of overall and specific Bisphenol-A migration of baby feeding bottles on the second assay with the simulants employed, expressed as concentration and standard deviation in mg of Bisphenol-A per kg of food simulant.

According to the results, overall migration was observed in all food simulants, especially in those called alternative fatty food simulants. Global migration assays results were below OML (60 mg/kg [2] and 50 mg/kg [8]). Although, OML is in agreement with the regulation, specific migration of other chemicals such as Bisphenol-A is unknown. For this reason, we evaluated both overall and specific migration. In addition, a second migration assay was done in order to evaluate the increase of specific migration along the reuse of the feeding bottles [10, 12].

After a first migration assay, Bisphenol-A was not detectable in any of food simulants and samples, while in the second migration assay, Bisphenol-A was observed in ethanol 15%, ethanol 95%, and isooctane. No migration was observed in water. The higher migration was obtained for the second migration assay with ethanol 95% and isooctane.

These results are in agreement with previous studies, which could be explained by effects such as cleaning, temperature, and sterilization with boiling water [10, 12, 20]. Our results demonstrate the importance of doing several migration assays for reusable products such as baby feeding bottles.

## 4. Conclusion

In this work, a fast, economic, simple, and accurate method for quantitation of Bisphenol-A migration from baby feeding bottles to food simulants using HPLC-UV-Vis was validated. One of the principal features of the developed method is the LOD 0.004 mg/kg and LOQ 0.010 mg/kg, which are enough to ensure compliance even with hardeners international regulation (0.05 mg/kg). Although other instrumental techniques reached lower LOD and LOQ, the validated method is easily accessible in most of routine analysis laboratories.

According to the migration results, this work shows that although the new baby feeding bottles have a Bisphenol-A migration lower than the limit established by actual legislation (SML 0.05 mg/kg), a higher specific migration was observed on a second migration assay using ethanol 95% and isooctane as food simulants. It demonstrates the necessity to carry out several specific migration assays for reusable plastic materials in contact with food to guarantee the health of consumers.

## Figures and Tables

**Table 1:** Precision and trueness of the HPLC-UV-Vis validated method.

Intraday %RSD (*n*=10)		Interday %recovery (*n*=3)			
Level (mg/kg)	Repeatability	Spiked level (mg/kg)	4th day	15th day	30th day
0.05	99.0	0.05	83.2 ± 4	86.1 ± 5	88.6 ± 3
0.3	96.0	0.3	83.7 ± 6	90.3 ± 3	95.7 ± 3
0.6	89.5	0.6	93.2 ± 4	94.8 ± 5	98.4 ± 1

**Table 2:** Results of overall migration and Bisphenol-A specific migration from 30 baby bottles in a second migration assay.

Migration assay	Water	Acetic acid 3%	Ethanol 15%	Ethanol 95%	Isooctane
Overall (mg/kg), *n*=3	2.6 ± 0.2	1.4 ± 0.2	4.8 ± 0.2	5.4 ± 0.3	7.1 ± 0.06
Specific (mg/kg), *n*=3	ND	<LOD	0.010 ± 0.001	0.115 ± 0.032	0.111 ± 0.001

ND = not detected; <LOD = lower than detection limit (0.004 mg/kg).

## Data Availability

The data used to support the findings of this study are available from the corresponding author upon request.
